# Recent Advances on the Bovine Viral Diarrhea Virus Molecular Pathogenesis, Immune Response, and Vaccines Development

**DOI:** 10.3389/fvets.2021.665128

**Published:** 2021-05-14

**Authors:** Anwar A. G. Al-Kubati, Jamal Hussen, Mahmoud Kandeel, Abdullah I. A. Al-Mubarak, Maged Gomaa Hemida

**Affiliations:** ^1^Department of Microbiology, College of Veterinary Medicine, King Faisal University, Al-Ahsa, Saudi Arabia; ^2^Department of Biomedical Sciences, College of Veterinary Medicine, King Faisal University, Al-Hofuf, Saudi Arabia; ^3^Department of Pharmacology, Faculty of Veterinary Medicine, Kafrelsheikh University, Kafrelsheikh, Egypt; ^4^Department of Virology, Faculty of Veterinary Medicine, Kafrelsheikh University, Kafrelsheikh, Egypt

**Keywords:** BVDV, genome, pathogenesis, mapping epitopes, immune evasion, immunotherapeutic, vaccines

## Abstract

The bovine viral diarrhea virus (BVDV) consists of two species and various subspecies of closely related viruses of varying antigenicity, cytopathology, and virulence-induced pathogenesis. Despite the great ongoing efforts to control and prevent BVDV outbreaks and the emergence of new variants, outbreaks still reported throughout the world. In this review, we are focusing on the molecular biology of BVDV, its molecular pathogenesis, and the immune response of the host against the viral infection. Special attention was paid to discuss some immune evasion strategies adopted by the BVDV to hijack the host immune system to ensure the success of virus replication. Vaccination is one of the main strategies for prophylaxis and contributes to the control and eradication of many viral diseases including BVDV. We discussed the recent advances of various types of currently available classical and modern BVDV vaccines. However, with the emergence of new strains and variants of the virus, it is urgent to find some other novel targets for BVDV vaccines that may overcome the drawbacks of some of the currently used vaccines. Effective vaccination strategy mainly based on the preparation of vaccines from the homologous circulating strains. The BVDV-E2 protein plays important role in viral infection and pathogenesis. We mapped some important potential neutralizing epitopes among some BVDV genomes especially the E2 protein. These novel epitopes could be promising targets against the currently circulating strains of BVDV. More research is needed to further explore the actual roles of these epitopes as novel targets for the development of novel vaccines against BVDV. These potential vaccines may contribute to the global eradication campaign of the BVDV.

## Introduction

BVDV is one of the most important viruses affecting bovine species throughout the world (1–4). It is a complex cocktail of two strains of the virus inducing various clinical syndromes of the affected animals (5). BVDV was first identified in the USA in a herd of cattle that suffered from acute gastroenteritis with high mortality rates (6). Since that time, BVDV represents one of the main viral pathogens of cattle in several regions across the globe (7–9). Some classical live attenuated and inactivated vaccines against BVDV are being in use for many decades for the immunization of the animals. Some of these vaccines usually trigger and induce the production of some levels of both humoral and cell-mediated immune responses (10). However, the emergence of new strains and variants hampered the efficacy of these conventional vaccines. Thus, the identification of some novel vaccines that match the new circulating strains in certain regions of the world would be of great impact on the contentious combating efforts for the BVDV. The eradication of the BVDV mainly depends on several factors including rigorous and continuous monitoring of the virus strains and variants, development of the most up-to-date diagnostic with high throughput ability to detect various strains and variants, and any potential new emergent strains or variants of the virus simultaneously as well as the development of homologous vaccines. We already mapped some novel neutralizing epitopes across the genome of BVDV, particularly the E2 gene. These epitopes could represent some unique targets for the production of novel vaccines against BVDV in the future. However, confirmation and validation of these potential epitopes require further studies.

## Morphology, Structure, and Classification of BVDV

**Figure 1:**
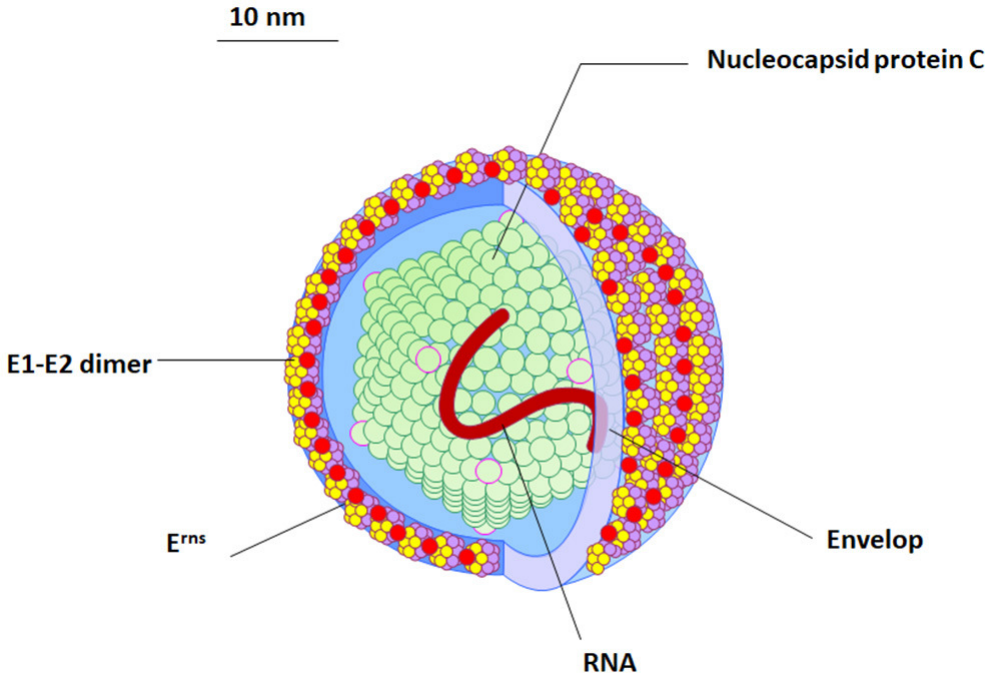
The morphology and structure of BVDV virus. An illustration of the BVDV particle showing the morphology and structure of the virus. Four structural proteins (Protein C, Erns, E1, and E2) are enclosing RNA (red). The outer viral protein coat contains important E1–E2 heterodimers which is required for the virus entry.

The BVDV particles are spherical to semi-spherical in shape (Figure 1). The virus particle consists of an outer bi-lipid layer envelope surrounding an electron-dense core as revealed by cryo-electron microscopy and negative staining electron microscopy (11, 12). There is some variation in the size of virus particles with a diameter of approximately 50 nm (range between 40 and 60 nm) for the majority of virus particles, but about 2% of the particles show a diameter of ~65 nm (11, 12).

The BVDV belongs to the genus *Pestivirus* in the family *Flaviviridae*, which includes other viruses affecting sheep and swine species (border disease virus and classical swine fever virus, respectively) (13). Both genotyping and serotyping are effective tools for variation mapping and classification of BVDV (14). Two genotypes of BVDV were identified based on their cytopathology and cell culture growing abilities in cell culture (BVDV-1 and BVDV-2). Meanwhile, there are two known biotypes within each genotype of the virus. The major effect of the BVDV infection in cattle is the reproductive, respiratory, and immunosuppression while, viral infection is rarely causing diarrhea and digestive tract problems (15).

Consequently, sequence relatedness became an essential parameter of species assignment (14). The HoBi-like virus was recently identified in Europe from the fetal bovine sera imported from Brazil (14). This virus was potentially classified as one of the atypical pestiviruses or belong to the BVD-3 serotypes (14).

## Genome Structure and Organization of BVDV

**Figure 2:**
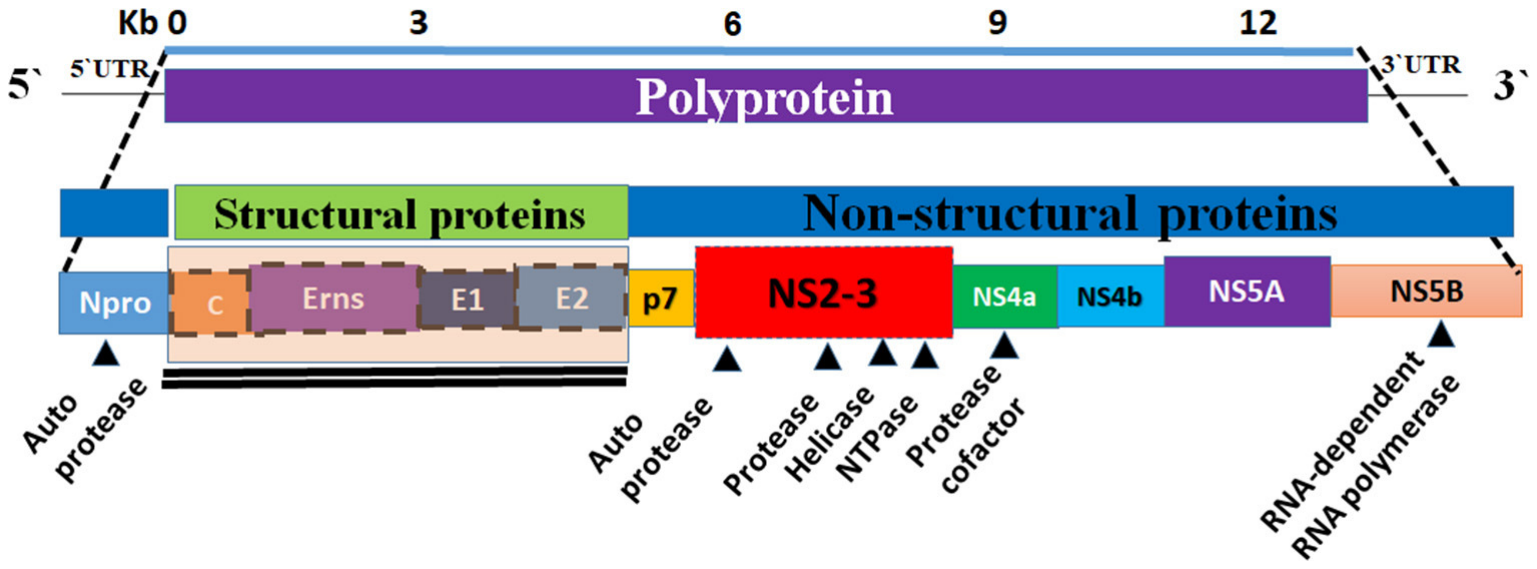
Graphical representation of the genome organization of BVDV. The BVDV genome is composed of a single strand of RNA (the bottom panel). The viral genome encodes a polyprotein (top panel). The central panel showing the composition of the viral genome (the structural and non-structural proteins). Eight non-structural proteins (Npro, p7, NS2, NS3, Ns4a, NS4b, and NS5b) and four structural proteins (C, Erns, E1, and E2) are encoded by polyprotein (middle and bottom panels). The non-structural proteins are encoding the viral proteases, helicase, NTPase, and RdRP.

The BVDV genome is ~12.3 kilo-bases (kb) in size and it consists of a single open reading frame (ORF) flanked by short 5′- and 3′-untranslated regions (UTRs) (16). The 5′-UTR of BVDV contains an internal ribosomal entry site (IRES) that functions to initiate the translation of a single polyprotein. The IRES is composed of 3 helices that contain two highly variable regions (17). The 3′-UTR contains conserved stem-loops instead of the poly-A tail and has sites for binding for several host cell microRNAs (16, 18). The ORF encodes a single large polyprotein that is post-translationally processed into four structural proteins and eight non-structural ones. The genome organization is as follows (NH2–Npro/C/Erns/E1/E2/p7/NS2/NS3/NS4A/NS4B/NS5A/NS5B–COOH) (Figure 2) (19). The BVDV ribosomal frameshifting is mainly due to the missing of one nucleotide in the Npro coding region, which occurs in the SD-1 strain of BVDV resulting in a reduction in viral RNA and viral protein in infected cells (20).

Three regions are usually targeted for genotyping of *Pestivirus* the 5′UTR, which contains intra-species and inter-species conserved motifs, and the Npro region, which is a unique region to Pestiviruses (14). Those two regions out of them were frequently used for genotyping, especially 5′UTR (14, 21–23). However, the third region is the coding region of E2 protein also showed high variability and was frequently used for genotyping of *Pestivirus*es (8). Based on partial or complete genome sequencing, BVDV-1 has been classified into at least 22 subtypes (BVDV-1a to BVDV-1v) (24) while BVDV-2 and HoBi-like virus divided into 4 subtypes (a to d) (25).

Another method based on the secondary structure of the 5′UTR palindromic region has been adopted for typing of *Pestivirus*. A software for palindromic nucleotide substitution (PNS) typing of *Pestivirus* was developed and used to type 543 sequences into 9 species in the genus *Pestivirus* (26). Similarly, PNS typing of 281 strains of BVDV-1 showed that it segregated into 15 genotypes (BVDV-1a to−1o) with 4 common PANs in the variable loci, V1, V2, and V3, of the 5′UTR that characterize BVDV-1 (27). However, PNS typing of 536 *Pestivirus* strains showed that 32 strains, that were isolated from a small ruminant with a clinical picture of border disease, were assigned to BVDV-1, BVDV-2, CSFV, and tentative BDV-2 (28). Homologous recombination has been reported to naturally occurs in members of Pestivirus, including BVDV-1 and−2, emphasizing the need to build genotyping on the sequence of multiple regions (29, 30). The results of genotyping usually agree with serotyping (14). The phylogenetic analysis was based on the 5′UTR and the E2 sequences of 30 Argentinean isolates of the BVDV. About 76% of these isolates were belonging to the BVDV1b however, the BVDV (1a, 2a, and 2b) were also detected in this study (31). Species of the *Pestivirus* usually showed some degree of antigenic relatedness, and the titer of neutralizing antibody in sera from infected/vaccinated animals against viruses belonging to the same species are several-fold higher than the titer against viruses from other *Pestivirus* species (14). Based on the virus neutralization test, there is some antigenic variability within the HoBi-like virus, and higher antigenic variability between the HoBi-like virus and BVDV-2, and even higher antigenic variability with BVDV-1 (23, 32, 33). A serosurveillance study on Hobi virus was conducted in Argentina (34). This study reported the detection of antibodies in sera of 12 large animals. The same study reported no or very mild antibody titers of other BVDV strains (BVD1a, BVDV1b, and BVD2) (34).

On the other hand, serotyping is not always constant with speciation based on host origin and clinical picture. Two *Pestivirus* isolates from sheep and goats with signs of border disease showed genetic and antigenic characteristics suggestive of a new species closer to the classic swine fever virus (35). Similarly, strains isolated from beef cattle and genotyped as BVDV-2a have shown a high ability to react with both anti-BVDV-1 and anti-BVDV-2 (36).

As the virus neutralization test (VNT) solely was not always sufficient for serotyping. To improve the performance of this serotyping approach, some monoclonal antibodies were also for differentiation of various BVDV serotypes and considered as diagnostic markers (14, 37). The level of cross-reactivity varies according to the targeted protein was reported using various monoclonal antibodies (38). For instance, cross-reactivity between BVDV-1/-2 and HoBi-like virus using anti-Erns or anti-NS2/3 monoclonal antibodies was higher than that using an anti-E2 monoclonal antibody (33). Genotyping by sequencing some key regions in the BVDV genome such as the 5′UTR is one of the most useful tools for virus identification (39). One of the best examples is the study conducted in China and identified that BVDV1b and BVDV1c are the predominant subgenotypes in some cattle populations in this region (39). Additional typing method based on the cytopathic effects of the virus growing on the infected cell culture. Accordingly, *Pestiviruses* were classified into two biotypes, cytopathic (CP), and non-cytopathic (NCP) strains (40). NCP-BVDV was further divided according to the exaltation of Newcastle disease virus (END) in certain cell cultures, for e.g., RK13, into two types END^+^ and END^−^ (41–43).

One of the limitations of antigenic characterization of BVDV is the antigenic diversity and cross-neutralization among isolates (44). A recent study employed a multivariate analysis for visualization of virus neutralization results to analyze the antigenic relationships between vaccine strains and some field isolates. Based on the demonstrated clustering patterns between the isolates the study concluded that BVDV-1 and BVDV-2 had the greatest antigenic differences (44).

Until recently, only four species were grouped in the genus *Pestivirus* including BVDV, CSFV, BDV, and unassigned *Pestiviruses* (26, 45, 46). Recently, Smith et al. proposed host-independent names for these species (*Pestivirus* A to D) and the addition of 7 new species (*Pestivirus* E to K) based on their sequence relatedness to *Pestiviruses* (Table 1) (13). According to ICVT, no change was introduced on the taxon of *Pestivirus* up to the time of writing this review (48). However, six viruses were reported from bat and rodents in China and proposed to form 6 new species based on their phylogenetic divergence (47). The sequence divergence of more than 25% with other *Pestiviruses* based on complete genome sequence was suggested as a criterion to assign separate species, as with the Brazilian strains of the HoBi-like virus that showed sequence similarity of 66.3 to 68.1% with representatives of Pestivirus species (49).

**Table 1:** Member of the genus Pestivirus, adapted from Smith et al. (13).

**Species name**	**Common name**	**Host**
Pestivirus A	Bovine viral diarrhea virus 1	Cattle, other ruminant, and pig
Pestivirus B	Bovine viral diarrhea virus 2	Cattle, other ruminant, and pig
Pestivirus C	Classical swine fever virus	Pig
Pestivirus D	Border disease virus	Sheep, other ruminant, and pig
Pestivirus E	pronghorn Pestivirus	Antelope
Pestivirus F	Bungowannah virus	Pig
Pestivirus G	Giraffe Pestivirus	Giraffe, cattle
Pestivirus H	Hobi-Like Pestivirus	Cattle, buffalo
Pestivirus I	Aydin-Like Pestivirus	Sheep, goat
Pestivirus J	Rat Pestivirus	Rat
Pestivirus K	Atypical porcine Pestivirus	Pig

*Several tentative species including Pestivirus from bat, rodent, sheep, and goat (13, 47)*.

## Processing and Maturation of the BVDV Polyproteins

BVDV encodes a single polyprotein that is post-translationally cleaved into four structural proteins (C, Erns, E1, and E2) (Figure 2) and 8 nonstructural proteins (Npro, p7, NS2, NS3, NS4A, NS4B, NS5A, NS5B). Table 2 summarizes the main aspects of BVDV proteins. The structural proteins can be classified as three envelope proteins (Erns, E1, and E2) and one capsid protein (nucleocapsid protein C). Among the structural proteins, C protein is the most abundant protein, followed by Erns, while E1 and E2 showed limited presence. However, on the surface of the virion, E2 is the most abundant surface protein and induces an immune response against BVDV, followed by Erns. All envelop proteins are produced as precursor protein Erns/E1E2, which passes two-step cleavage reactions to produce the free envelope proteins Erns, E1, and E2. Both E1 and E2 contain transmembrane domains, while Erns is only anchored to the membranes, which also allows for the secretion of Erns (65, 66). Compared with MDBK cells, lipids of BVDV contain more cholesterol, sphingomyelin, and hexosyl-ceramide and fewer glycerol-phospholipids with unknown mechanisms of lipid sorting. Cholesterol and sphingomyelin were shown to be important for BVDV entry (11).

**Table 2:** The structure and functions of the BVDV proteins.

**Protein**	**Structure**	**Function**	**Notes**	**References**
Npro		• Suppress IFN I • Auto-protease	• Unique to Pestivirus • Bind to cellular S100A9 protein	(50)
Core (C) protein	Highly basic	Bind to RNA, 14 Nucleotides/C-protein molecule		(51)
Erns	• 42–48 kDa • C-terminus folds into an amphipathic helix • Highly glycosylated • belong to T2 family of endoribonucleases	• RNase activity for degradation of ds, ssRNA • Inhibit IFN-I production by removing PAMP in intracellular compartments	Unique to pestivirus	(50–53)
E1	25 kDa	Fusion during entry	Co-localize with autophagy marker light chain (LC)-3	(11, 51, 54)
E2	• 55 kDa, 373 amino acids • Three domains, domain I and II (epitopes), domain III (anchor)	• Attachment protein • Suppress complement mediated cell lysis and DNA fragmentation	• Form homodimer, E1–E2 heterodimer • Co-localize with autophagy marker LC-3 • Posses neutralizing epitope • Variability, use for phylogeny	(8, 11, 25, 55, 56)
P7	7 kDa	Ion channel activity, has a role in assembly	Found as individual or part of E2–P7 precursor	(51)
NS2-3		Virion morphogenesis		(40)
NS2	• 450 amino acids • Hydrophobic N-terminus with up to 7 transmembrane segments	Cysteine-auto-protease	Cellular DNAJC14	(40, 51)
NS3	80 kDa 683amino acids immune-dominant part located between aa205 to 549	• Serine protease • RNA helicase • NTPase	• Function with viral NS4A as cofactor • Highly conserved • Immunogen	(40, 57)
NS4A	66 amino acids, first 1/3 is Hydrophobic, last third is acidic	Virion morphogenesis	Require NS3, Co-localize with ADAR in cytoplasm	(51, 58)
NS4B	• 38 kDa • Highly hydrophobic	Induction of autophagy, scaffold for viral replication complex	Single mutation (Y2441C) change from cp to None-CP type. Co-localize with autophagy marker LC-3	(59, 60)
NS5A	56–58 kDa, Phosphoprotein	Component of viral replicase	Bind to bovine NIK- and IKKb-binding protein (NIBP), Co-localize with NIBP on the endoplasmic reticulum. Co-localize with autophagy marker LC-3	(61, 62)
NS5B	Four domains fingers, palm, thumb and unique N-terminal domain	RNA-dependent RNA polymerase	Highly conserved, certain few mutations in the binding pocket reduce the catalysis and fidelity	(63, 64)

**Figure 3:**
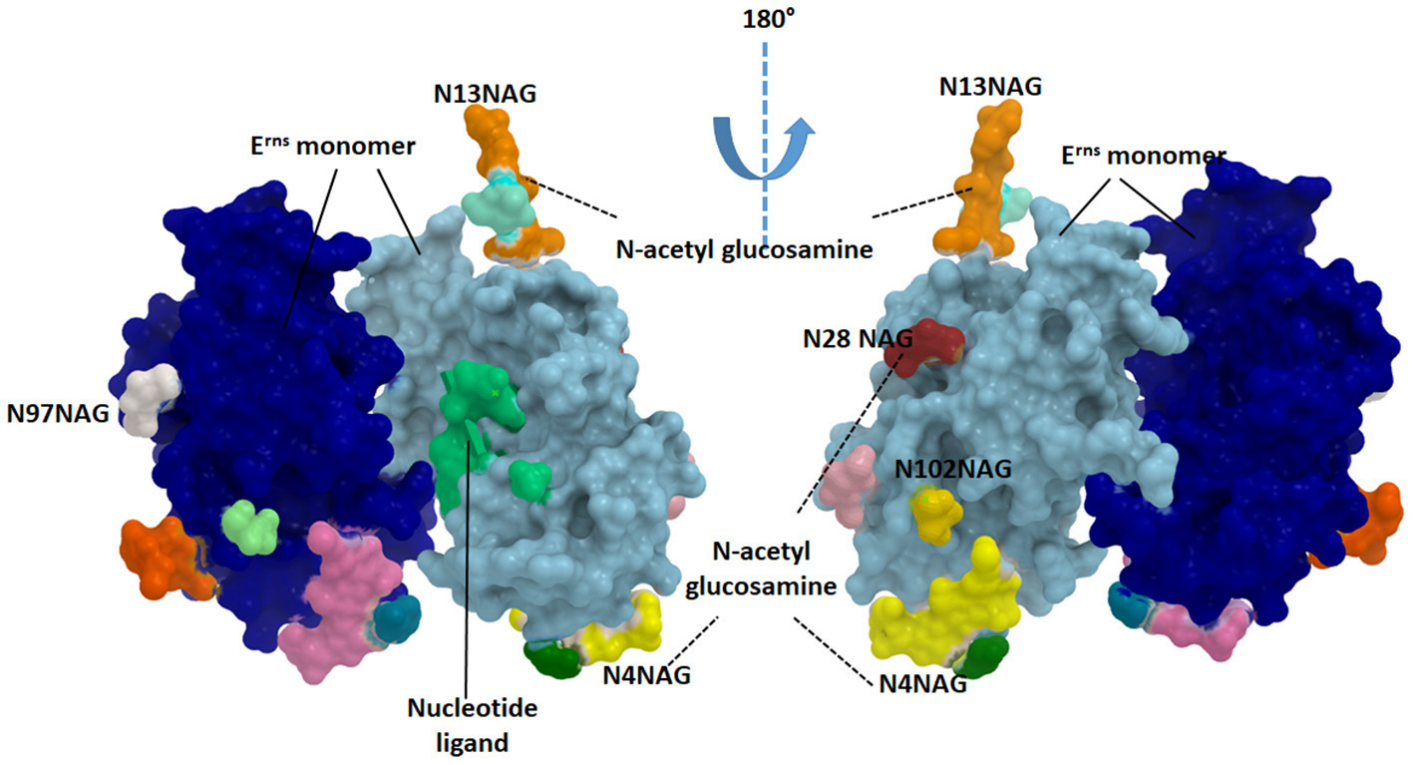
Structure of the catalytic domain of envelope E^rns^ glycoprotein. Each monomer is showing in blue and cyan colors. The glycosylated sites are provided in green (N4NAG), orange (N13NAG), red (N28NAG), white (N97NAG), and yellow (N102NAG). The figure was derived from the structure deposited in the protein data bank (PDB ID 4dvn).

The Erns protein is one of the unique proteins in the *Pestiviruses'* genome which can bind with nucleotide substrates and with evidence of ribonuclease activity (Figure 3). This protein was found to be highly similar in structure to T2 ribonucleases from plants and fungi (67). The majority of Erns protein is secreted into the infected cell culture media (68). Erns is believed to make heterodimer very early during the virus replication which suggests its roles in the virus attachment to the target cells (68).

Erns released from the infected cells interfere with immune response by the degradation of the circulating nucleic acids (69). Erns shows RNase activity in the intracellular compartment, thus preventing IFN production by degrading RNA and removing the resistant pathogen-associated molecular pattern (PAMP), thus maintaining the appropriate milieu for persistent infection (52). As an RNAse protein, Erns is a glycosylated protein carrying several N-acetyl glucosamine molecules (Figure 3). Erns activity is not confined to bovine cells. Extracellularly added Erns was shown to be uptaken into bovine turbinate cells, probably by clathrin-dependent endocytosis, and to remain active for a long time after been engulfed. Degradation of viral RNA takes place in endosomal compartments before reaching cytosol (70). The Erns protein belongs to the T2 family of endoribonucleases that preferably cleave ssRNAs. However, monomeric Erns showed the ability to cleave dsRNA and RNA in DNA/RNA, methylated RNA/RNA hybrid (53).

**Figure 4:**
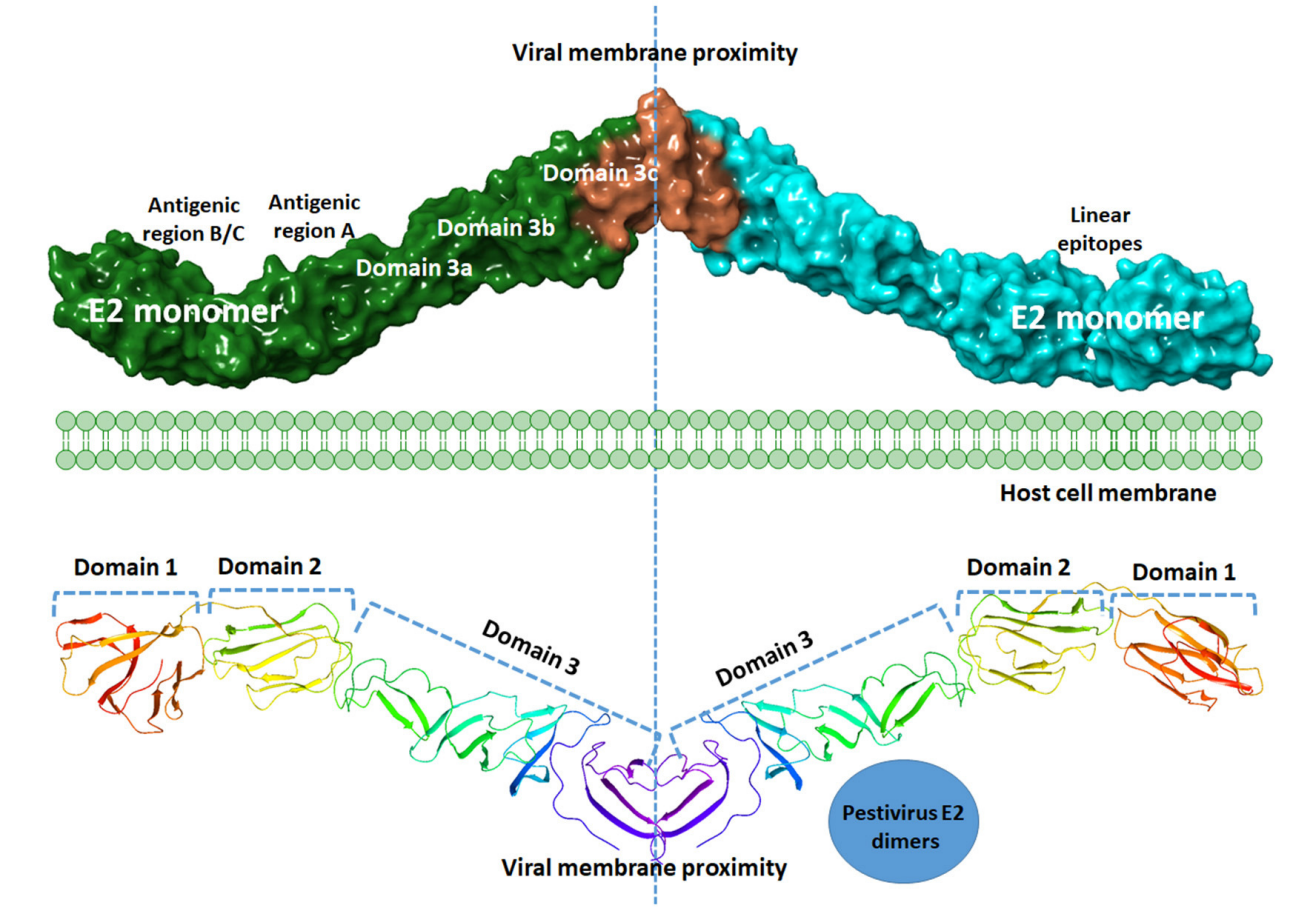
Graphical representation of BVDV E2 dimers. A model of the graphical representation of the E2 dimers. Cartoon (upper panel) or surface representation (lower panel) are provided. The domain contents of each monomer, antigenic sites, and the subdomains are highlighted. Each E2 monomer contains three domains, domain 1, 2, and 3. Domains can be divided into subdomains 3a, 3b, and 3c. Three regions of linear epitopes comprising antigenic regions A, B, and C. E2 glycoprotein is thought to share in the membrane fusion process during virus entry, yet the exact mechanism is to be investigated. The figure was derived from the structure deposited in the protein data bank (PDB ID 4jnt).

The surface of the BVDV mainly consists of the E2–E2 homodimers and the E1–E2 heterodimers (11, 71). This heterodimer is the most important protein for virus fusion with the host cells (72). However, due to the absence of hydrophobic core and lacking fusogenic sequences, the E2 function in virus fusion was not proposed and the functionally uncharacterized E1 might carry out this role (73). The main function of the E2 glycoprotein (55 kDa) is the attachment to the host cell by forming the homodimers beside the mentioned heterodimerization with E1 (11). The E2 ectodomain consists of three domains with a total span of 140A with no known fusion motifs (Figure 4). Domain I and II are Ig-like domains with 90 and 78 amino acid residues, respectively. Domain III consists of 175 amino acids that form three β-sheet modules (IIIa-IIIc) (55). The c-terminus contain a single-span transmembrane anchor that retained the E2 glycoprotein in ER (74). Post-translationally processed E2 contains four glycans and eight disulfide linkages. A ninth disulfide link is used to form an end-to-end homodimer, a linkage that explains the need to low pH activation to initiate fusion (55).

Some recombinant E2 based baculovirus vaccines were produced commercially and showed promising trends in bosting the immune-response against BVDV in combination with the inactivated BVDV in the goat model (75). Some monoclonal antibodies against E2 of the CSFV fulfilled the DIVA concept which is an important approach to differentiate between the infected and vaccinated animals (76).

**Figure 5:**
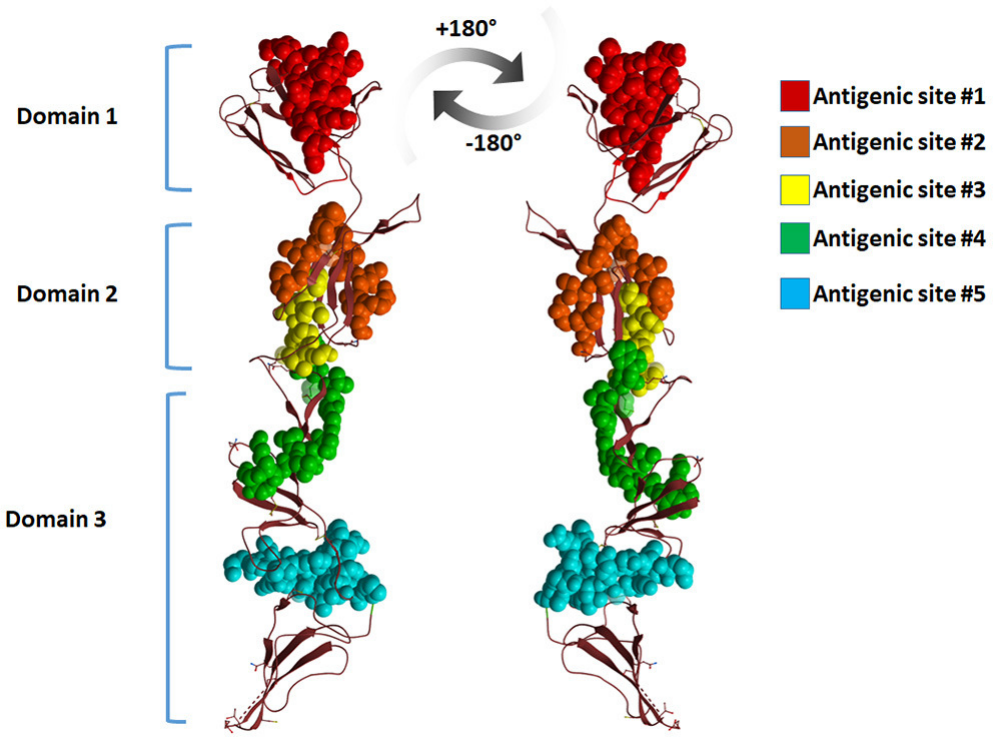
Cartoon representation of BVDV-E2 glycoprotein monomer showing the antigenic sites predicted by EMBOSS antigenic prediction tool. The top five antigenic sites were given in spheres with different colors. The figure was derived from the structure deposited in the protein data bank (PDB ID 4jnt).

Mapping the antigenic epitopes within the E2 of BVDV-1 and -2 showed the presence of type-specific epitopes in a context of comparable antigenic structure (77). It consists of four epitopes, two in each of domain I and domain II (Figure 4). While, domain III showed no epitopes, with IIIc as the most conserved part of the E2. Most of the escaping mutants were mapped to one face of the E2, suggesting that this is the exposed face (55). For further investigation of BVDV E2 glycoprotein epitopes, the sequence of E2 protein was mapped by EMBOSS antigenic predictor tool (78). About 17 antigenic sites were predicted with six or more amino acids count (Table 3). Close inspection of the delivered sites of predicted intensity reveals the potential inclusion of domain III (Figure 5). Previous reports showed that domains I and II are the only parts of E2 that are exposed to the immune reaction (55). However, based on the EMBOSS antigenic prediction tool results, the potential finding of antigenic sites in E2 domain III gives new insights into the structure and function of E2 protein. It was predicted that only domains I and II were exposed to the immune reactions owing to their potential presence on the surface of the virus, a property that is lacking in the 3^rd^ domain. By the presence of epitopes in domain III, the whole structure of E2 might be then exposed to the virus surface and might contribute to the immune response against the virus as well as other functions e.g., participation in membrane fusion and virus entry process.

**Table 3:** The predicted antigenic epitopes in BVDV E2 glycoprotein.

**#**	**Score**	**Sequence**	**Residue range**	**Length**
1	1.156	YLAILHTRALPTSVVFKKL	64 –> 82	19
2	1.143	FGLCPCDAKPIVRG	100 –> 113	14
3	1.135	FQMVCPIG	125 –> 132	8
4	1.134	VAIVPQGTLKCKIGKTTVQVIAM	246 –> 268	23
5	1.123	WTCVPGDQLLYKG	186 –> 198	13
6	1.121	YRLVDST	233 –> 239	7
7	1.117	LHNCILG	177 –> 183	7
8	1.115	ATTVVRTY	148 –> 155	8
9	1.112	LMYLQRC	52 –> 58	7
10	1.104	TVSCTS	136 –> 141	6
11	1.102	PMPCRPYEIISSTACTFN	274 –> 291	18
12	1.1	GLPHYPIGKCKL	216 –> 227	12
13	1.1	IESCKWCGYQ	201 –> 210	10
14	1.091	DTMVIAWC	40 –> 47	8
15	1.074	KPEFSYAIA	4 –> 12	9
16	1.074	SYFQQYM	306 –> 312	7
17	1.071	PFPHRQGCITQ	160 –> 170	11

*The antigenic sites were sorted by descending score of the EMBOSS antigenic tool*.

Persistently infected animals (PI) usually developed during embryonic developments during the first 125 days of gestation (9). When these calves are born, they become a major source of infection and virus spread by secreting a large number of viruses in their body secretions and excretions (79).

Comparing the genome sequences of the BVDVs from severe acute infections and PI showed that the mutation rate was higher in viruses from PI (80). Similarly, an increase in mutation rate was reported after serial transmission in pregnant bovine, ovine (81), caprine (82), and swine (83). Certain amino acid substitutions appear after transmission from species to species (81–83). Mutation tends to concentrate in the coding regions of the structural proteins, especially the E2 protein (80–84). Several studies suggest the occurrence of natural recombination between BVDV genomes. It is usually associated with PI and concomitant immunotolerance, a situation in which mixed infection and consequent recombination may occur (30, 85). Similarly, recombination of BVDV genome with cellular RNAs in PI animals was incriminated as a mechanism that gives rise to mutant BVDV that induces the lethal mucosal disease (86). BVDV recombination was reported to occur even in the absence of a translation of viral proteins or even active viral RNA-dependent-RNA-polymerase (86, 87).

## BVDV Replication Cycle and Pathogenesis

The outcomes of the BVDV infection in susceptible animals are mainly dependent on the breeding status of the animals. BVDV infection in pregnant cattle may result in several syndromes, including early embryonic death, teratogenic effects on the fetus, and the development of persistently infected animals (PI) (5). The PI plays an important role in the sustainability of the BVDV in the environment and the transmission of the virus from animal to animal as well as from one herd to another. The PI animal continues to shed the BVDV in its body secretions and excretions throughout its life, posing a major risk to other animals within the herd and in close contact herds as well (6, 19, 88). Another concern about the PI animals is they cannot be identified easily throughout the animal's life, especially immediately after birth due to the over masking effect of the antibodies from the colostrum, as described below in more details (5).

The susceptibility of cattle to the BVDV infections is mainly associated with some loci on cattle chromosome 2 (BTA2) and 26 (BTA26) (89). These regions showed their substantial contribution to the animal susceptibility to PI-BVDV infections (90). This genetic predisposition may relate to individual cattle rather than to a specific breed. This could be supported by the significant difference in the replication of BVDV in cell culture derived from various breeds and individuals animals within-herd (91).

Cross-species transmission is a highly important phenomenon that may lead to the establishment of a new reservoir for the virus which makes the control and eradication is too difficult in its natural reservoir. The transmission from PI cattle to goats was reported up to two generations of goats (80, 82, 83).

The CD46 molecule act as a BVDV receptor during virus entry. Some studies showed that the NADL-BVDV strain was able to spread from infected to susceptible cells via a CD46-independent mechanism (92). Despite the frequent association, BVDV-E2 binding to the CD46 receptor was not required for BVDV uptake, suggesting the involvement of other cellular proteins (93). Upon uptake into ovine cells (SFT-R), the NCP-BVDV enters the eclipse phase of replication that takes 8–9-h. The positive and the negative strands of the viral RNAs first appear at 4-h post-infection (hpi). In those ovine cells, the complete replication cycle takes 10–12-h, and infectious BVDV particles first appear intracellularly and extracellularly at 8 and 10 hpi, respectively. Virus titer and positive-strand RNA reach a peak at 16 hpi. NS3 and E2 proteins appear at 6 and 7 hpi, respectively, while NS2-3 gradually accumulated thereafter (94).

Replication of the NCP-BVDV is regulated via the mechanism of cleavage of NS2-3, which is performed by NS2 protease activity that requires DNAJC14, a limiting cellular cofactor. Cleavage of NS2-3 led to the generation of the NS3, an essential part of the replicate (40). During replication, the 5' UTR of BVDV stalls the 5'−3' exoribonuclease (XRN1) enzyme and repress its activity resulting in a significant increase in the half-life time of many normally short-lived cellular mRNAs (95).

The gene expression and genome protection against transposon and viruses are among many functions played by non-coding RNA, such as long ncRNA (lncRNA), short ncRNA (sncRNA), microRNA (miRNA), and transfer RNA halves (ts-RNA) (96, 97). Studies on the expression of lncRNA in BVDV infected MDBK cells revealed the enrichment of several pathways particularly related to immune response such as the T-cell receptor, TNF, Jak-STAT, apoptosis, Ras, NOD-like receptor, NF-κB, ErbB, and fatty acid biosynthesis (98, 99). Similarly, analysis of the expression of circular RNA (circ-RNAs) in BVDV infected MDBK cells suggest their involvement in the regulation of cell proliferation and apoptosis (100). An additional mechanism used by BVDV to indirectly control cellular transcriptome is the sequestration of small non-coding RNA, such as miR-17 and let-7, that is required for BVDV replication, as shown in SK-6 cells (18). The expression levels of other microRNAs, like bta-miR-423-5P and bta-miR-151-3p and some transfer RNA halves (ts-RNAs) in calves sera, differ significantly as a function of BVDV infection and time post-inoculation (97, 101). RNA interference by short interfering RNA (siRNA) that targets the 5'UTR and the envelop glycoproteins Erns, E1, and E2 coding regions induce a moderate reduction in viral titer, antigen, or RNA copy numbers in MDBK cells infected with BVDV-1 (102).

## BVDV-Induced Immune Response and Immune Dysfunction

The persistent infection and dysregulated immune response are two major consequences of BVDV infections (103, 104). Protective immunity after natural infection with BVDVs is characterized by the activation of both virus-specific humoral and cellular immune responses (105). Due to their central role in guiding humoral and cell-mediated immune responses, CD4+ T helper cells, mainly targeting the NS3 and E2 proteins, are key players in the development of protective immunity against the virus (106). In opposite to the depletion of CD8+ T cells, depletion of CD4+ T helper cells was associated with higher blood viral load, prolonged viremia, and virus secretion via the nasal route (107). After B cell activation, neutralizing antibodies are detectable, starting on day 14 of the infection (105). The most effective neutralizing antibodies mainly target the surface protein E2, whereas antibodies specific to the Erns possess less neutralizing activity (108). While some antibodies target the E1, the main structural protein of BVDV (C) does not induce B cell activation and antibody production. Also, the non-structural protein NS2-3 induces a strong antibody response (108). Several studies investigated the impact of the virus biotype on the course of the immune response against BVDVs. Comparative analysis of the immune response to the NCP and CP biotypes of BVDV suggests a higher potential for NCP-BVDV to induce humoral immunity, while infection with CP-BVDV resulted in a better cell-mediated immunity (88, 109, 110).

Several studies have been recently conducted to identify the mechanisms behind BVDV-induced immune dysregulation (110–114). BVDV-induced immunosuppression has been identified in naturally infected animals, with both transient or persistent infection, as well as after experimental BVDV infection (103). Immunosuppressive effects of BVDV include changes in the immune cell composition, and altered immunophenotype of leukocytes, and several defects in immune cell function, resulting in increased disease severity of secondary infections with other pathogens. In experimentally infected cattle, BVDV infection was associated with significant changes in the bovine leukogram with reduced numbers of total leukocytes, neutrophils, lymphocytes, and platelets (115–117). The functions of both myeloid and lymphoid cells are affected by BVDV. Functional analysis of neutrophils of the PI animals revealed reduced phagocytosis capacity and decreased reactive oxygen (ROS) production, compared to neutrophils from healthy animals (118). The inhibitory effect of BVDV on neutrophils has been found strain-specific (119). Although all BVDV strains (including CP and NCP-BVDV) induced a significant decrease in the expression of the cell adhesion molecules CD18 and L-selectin on neutrophils and impaired their *in vitro* ROS and neutrophil extracellular traps (NET) activity, only CP-BVDV reduced the phagocytosis function. In contrast, only NCP-BVDV enhanced CD14 expression on neutrophils and improved their chemotactic activity (119). A recent study evaluated the impact of supernatant collected from macrophages infected with different BVDV strains on macrophage inflammatory response and lymphocyte apoptosis. The results of this study revealed a role for macrophages-secreted mediators in the immune dysfunction associated with highly virulent NCP-BVDV (62). An essential role of danger-associated molecular patterns (DAMPs) and danger-sensing protein complexes in the dysregulated immune response to BVDV infection has been recently reported (120). A recent experimental infection study of bovine macrophages indicated the ability of CP-BVDV-1 to activate the danger-sensing multi-protein complex, the inflammasome, in a caspase-1 dependent manner, resulting in IL-1β secretion with increased viral replication (120). The role of the DAMP S100 protein A9 (S100A9), which induces its effect via toll-like receptor (TLR)-4 / MyD88 signaling, in the BVDV-induced immunosuppression, has been recently reported (50). The results of this study revealed that *in vitro* BVDV replication was enhanced by inhibiting S100A9 protein expression in BVDV-infected cells using siRNA, while overexpression of S100A9 enhanced the virus-induced type-I IFN production. The strong interaction between S100A9 in infected cells and the Npro protein of BVDV suggests a role of this interaction in reducing the type-I IFN response by reducing the availability of S100A9 protein (50).

BVDV also showed inhibitory effects on several adaptive immune cell functions. Polyclonal mitogenic stimulation of bovine lymphocytes from infected animals induced a reduced proliferative response, as compared to cells from healthy animals (118, 121). A recent *in vitro* infection model reported the immunosuppressive capacity of BVDV on bovine peripheral blood mononuclear cells (PBMCs) (114). Bovine PBMCs from BVDV-naturally infected animals were more susceptible to BVDV infection with a higher apoptosis response, compared to cells from naive animals. In the same study, *in vitro* infection with NCP-BVDV failed to induce the expression of cell surface markers related to antigen presentation function (114). This biotype-specific immunosuppressive effect on the adaptive immune response has also been reported in a recent study that investigated the effect of infection with CP, or NCP-BVDV strains on levels of total serum IgG, IgG1, IgG2, BVDV neutralizing antibodies, and total white blood cell count (113). While the infection with CP-BVDV resulted in an early (d7 pi) decreased levels of neutralizing antibodies and leukocyte numbers, the infection with NCP-BVDV induced polarization of the immune response toward the Th1 response with the production of more antibodies of the IgG2 isotype (113).

Some earlier studies suggested a role for the higher suppressive effect of NCP-BVDV on interferon response, compared to CP-BVDV, in the establishment of persistent infection with NCP but not CP-BVDV (122). However, recent studies identified the induction of a significant IFN-alpha (IFN-α), IFN-β, and IFN-γ response in the PI fetuses after *in utero* infection with NCP-BVDV (123, 124). This is also supported by another study on naturally infected cattle, showing that IFN signaling is not completely inhibited in PI cattle (125). Similarly, the higher frequency of cells expressing MHC class I and II molecules in liver tissues from the PI fetuses, compared to control fetuses, indicates the induction of an immune response to NCP-BVDV infection (126). The reason behind the failure of the immune response to clear the virus and the mechanism through which BVDV-PI is established, therefore, still to be elucidated. A recent study identified different immune responses of fetal lymphoid organs to transient and persistent BVDV infections (127). The PI fetuses showed reduced expression of several genes involved in the innate immune response and antigen presentation to adaptive immune cells; transiently infected fetuses upregulated several innate immune response genes in their thymuses. Also, several adaptive immune response genes were downregulated in PI fetuses. The study suggested a role for the suppressed innate and adaptive immune responses in the developing lymphoid organs in the persistence of the BVDV in the PI animals and, on the other hand, a role for the upregulation of the innate immune response genes in transiently infected fetuses in virus clearance from these animals (127). Bovine embryonic cells showed the ability to take the function of immune cells by recognizing and responding to BVDV infection through the upregulation of genes encoding for INFα and TLR7, which are involved in inflammatory and immune responses (128).

## BVDV Immune Evasion Strategies

Many viruses including BVDV use several unique immune evasion strategies to hijack the host immune response to ensure successful viral replication and spreading from one host to another. These strategies include the adaptation of several viral survival strategies, the “hit & run” approach, and viral persistence (122). These strategies favor the virus replication and spreading. On the other hand, these strategies reduce the values of the currently used diagnostic assays to identify the newly emerged strains of the virus. Furthermore, these evasion strategies will favor the emergence of new variants and strains of the virus, which may have negative impacts on the currently used BVDV vaccines in the protection of the animals at risk. The milestone of the BVDV infection among a certain population of animals is the establishment of innate immune tolerance in the pregnant animals to produce an immune tolerant and PI animal after birth (129).

Although the viral RNAs induce the IFN production and synthesis, the viral-Erns suppress the IFN production pathways triggered earlier by the viral RNAs (122, 130). The Npro induces degradation of the IRF3 (essential IFN activation factor) during the virus replication in cell culture (130). Both the Npro and the Erns act as an IFN antagonist in a non-redundant manner in the cell culture of the CP-BVDV and the NCP-BVDV strains (130). Although the NCP-BVDV strains efficiently cause evasion of the innate immune response in the affected animals (124), they can induce IFN-γ production during the acute phase of infection, especially in the PI animals (124). This indicates, that despite the marked inhibitory effects of BVDV infection on the IFN production in the affected host, it does not alter or suppress their actions (130). Another unique BVDV immune evasion strategy is the “self” and “non-self” alteration to the IFNα/β pathways (131). This selective phenomenon enables the BVDV to establish the persistence of infection in the affected animals, which maintains the circulation of the virus among a certain population of animals (131).

## Recent Advances on BVDV Vaccination and Immunotherapeutic Strategies

Due to the significance of PI animals for spreading the infection, the target of vaccination strategies against BVDV mainly focuses on fetal protection, in addition to preventing clinical disease and virus-induced immune dysregulation. Control vaccination programs against BVDV involved using different types of live attenuated, killed, and recombinant vaccines (132–136). Although several scientific reports suggested their ability to prevent the clinical BVDV manifestations in cattle, live attenuated and killed BVDV vaccines differ in their safety for the vaccination of different cattle populations. Different live attenuated vaccines have been developed and widely used against BVDV, usually resulting in strong humoral and cell-mediated immune responses with solid fetal protection (133, 137). Due to its higher safety, being not able to infect the fetus and establish a persistent infection, CP-BVDV was used for the development of the most recent attenuated vaccines. On the opposite, as they can cross the placenta and infect the fetus, live attenuated vaccines based on NCP-BVDV are generally not recommended for the vaccination of pregnant animals. However, recent attempts to overcome the safety problem of NCP-BVDV involved the development of a mutant virus after deleting the Npro gene and inactivating the endoribonuclease activity of Erns (112, 137). Although some reports indicated that the mutated virus can induce a strong immune response without crossing the placenta (112, 113, 137), a recent work, however, reported the ability of the vaccine virus to cross the placenta (138). This indicates the need for further studies for evaluating the safety of this type of vaccine.

The different adverse effects associated with live attenuated vaccines, including the intrauterine infection of pregnant animals and immunosuppressive effects of the vaccine virus, have prompted the need for safe killed vaccines that can be given at any age and stage of pregnancy (139–141). Although they are safer than live attenuated vaccines, killed vaccines have lower immunogenicity and need therefore to be injected several times with slowly developing immunity. In a recent report, vaccination with different killed BVDV vaccines failed to induce cross-protective antibody response against all used virus strains (142, 143). Although the application of killed vaccines is associated with high antibody titers, the effectivity of the induced cell-mediated immunity is variable (144, 145). Fetal protection after vaccination with killed vaccines varies from incomplete to satisfactory (141, 146, 147).

To enhance the reduced immunogenicity of killed vaccines, several different powerful adjuvants have been used to improve the immune response after vaccination. A recent work evaluated the effectiveness of using immunomodulatory adjuvants in the BVDV vaccine design. The administration of a subunit vaccine formulated of the BVDV type-2 E2 protein with a novel adjuvant containing a mixture of a TLR 3 agonist, poly (I:C); an innate defense regulatory peptide; and water-soluble polymer, poly[di(sodium carboxyl atoethyl phenoxy)]-phosphazene (PCEP) induced the development of a robust immune response. In addition to inducing a strong humoral immune response, the vaccine resulted in cross-presentation with the development of both virus-specific CD4+ and CD8+ T-cell-mediated immune responses (148). For achieving a balance between vaccine safety and immunogenicity, combined administration of killed and live attenuated BVD vaccines has been recently suggested to induce reproductive protection in cows (149). The potential advantage of a DNAprime–protein boost vaccination approach has been recently demonstrated in mice primed with a plasmid encoding the E2 protein and boosted with adjuvant recombinant E2 protein. The combination of a DNA prime with protein boost vaccination was effective in eliciting high neutralizing antibody titers and cellular helper and cytotoxic immune responses against the virus (150). An earlier study showed the cross-protection between the type-1 and type-2 BVDVs (151). Vaccination of some female cattle with the bivalent MLV vaccines from both the BVDV and the BoHV-1 was linked to some undesired reproductive problems, especially abortion (133, 152). An interesting study recently developed a quadrivalent recombinant vaccine to overcome the side effects of the bivalent vaccines (BoHV-1 and the BVDV) (153). This study showed superior results of the newly developed quadrivalent vaccine with much more potent neutralizing activities against both viruses. It also showed the cross-protection among the two types of the BVDV with minimal reproductive failure compared to the other commercially available vaccines (153). A further recent work reported the development and efficacy of the first targeted subunit vaccine against BVDV (154). The vaccine was based on the fusion of the BVDV structural protein E2 with a single-chain antibody for targeting the E2 antigen to the major histocompatibility complex (MHC) class II molecules on antigen-presenting cells. The developed subunit vaccine induced a rapid and sustained BVDV-specific neutralizing antibody response in cattle (154). The immunogenicity of a recombinant *Lactobacillus* vaccine (using *Lactobacillus casei* strain W56 as antigen carrier) constitutively expressing BVDV E2 protein fused with cholera toxin B subunit as an adjuvant has been recently evaluated in a mice model (136). The study reported the induction of protective mucosal, humoral, and cellular immune responses in vaccinated mice and suggested employing the used strategy for vaccine development against BVDV (136).

Several studies have been conducted aiming at the development of novel anti-BVDV therapeutic strategies. Based on their advantageous small size and stable chemical structure, a recent approach investigated the therapeutic potential of newly developed single-domain antibodies (Nanobodies) against the nonstructural protein 5 (NS5B) of BVDV, which play an essential role in viral replication (155). *In vitro* analysis confirmed the interaction between the NS5B-nanobody and the BVDV NS5B protein, resulting in a marked suppressive effect on BVDV replication (155). A further therapeutic approach against BVDV infection proved the anti-BVDV activity of a biologically active recombinant bovine IFN-lambda (IFN-λ) (156). Systemic administration of IFN-λ to cattle experimentally infected with BVDV induced a systemic type-I IFN response, prevented BVDV replication and the development of the clinical disease, and enhanced the humoral immune response against the virus (156). Although these novel therapeutic strategies may contribute to BVDV control in individual animals, they are difficult to be implemented in commercial animal herds.

## Maternal Immunity and Vaccination of Newborn Animals Against BVDV

The colostrum uptake supplies newborn calves with maternal antibodies, which prevent BVDV infection during the first weeks after birth (157). Maternal antibodies-mediated protection may, however, last for up to 9 months. The duration of protection in the newborn animal depends on the decrease in virus-specific antibodies in calf serum, which is mainly affected by the initial titer of colostrum's maternal antibodies (157). Although maternal antibodies are important for passive immunity and early protection, they may interfere with mounting active immune responses in vaccinated newborn calves (132, 158–162). Upon the vaccination of seven weeks-aged calves against BVDV, a T-cell mediated immune response developed despite the presence of circulating maternal antibodies (163). A recent BVDV vaccination study suggested a significant impact of virus biotype; method of attenuation, presentation, and use of adjuvant on the immune response of colostrum deprived calves and recommended the consideration of these variables when vaccinating newborn calves (111). The combination between a modified-live BVDV vaccine and the administration of some injectable trace minerals resulted in an enhanced immune response (titer of neutralizing antibodies) and improved health status of beef calves challenged with BVDV2 (164).

## Future Directions

Although BVDV reported for more than 6 decades, it is still one of the main common viral threats to the bovine industry. Despite the great research ongoing progress on BVDV from different aspects including molecular biology, pathogenesis, immune response, and control measures, many aspects need further studies. The continuous emergence of new variants and strains of the BVDV may hamper the efficacy of the currently available diagnostic assays and vaccines. Exploring the functional activities of the mapped neutralizing epitopes within the BVDV-E2 gene may help in the development of effective vaccines against viral infection shortly. Searching for new potential reservoirs for the BVDV is one of the understudied research lines. Identification of new BVDV reservoir/s may help in minimizing the spillover of the virus from these unknown hosts to the bovine and ovine species. Further substantial efforts are needed to combat the emergence and spread of BVDV to eradicate such an important virus from various regions across the world.

## Author Contributions

AA-K, JH, MK, MH, and AA-M prepared the original draft and revised the manuscript. AA-M acquired funding. All authors have read and agreed to the published version of the manuscript.

## Conflict of Interest

The authors declare that the research was conducted in the absence of any commercial or financial relationships that could be construed as a potential conflict of interest.
